# TSPO PET Identifies Different Anti-inflammatory Minocycline Treatment Response in Two Rodent Models of Epileptogenesis

**DOI:** 10.1007/s13311-020-00834-5

**Published:** 2020-01-22

**Authors:** Bettina J. Wolf, Mirjam Brackhan, Pablo Bascuñana, Ina Leiter, B. Laura N. Langer, Tobias L. Ross, Jens P. Bankstahl, Marion Bankstahl

**Affiliations:** 1 Department of Nuclear Medicine, Hannover Medical School, Carl-Neuberg-Str. 1, 30625 Hannover, Germany; 2 Department of Pharmacology, Toxicology and Pharmacy, University of Veterinary Medicine, Hannover, Germany; 3 Present Address: Department of Pathology, Section of Neuropathology, University of Oslo and Oslo University Hospital, Oslo, Norway; 4 Present Address: Institute of Neuroanatomy and Cell Biology, Hannover Medical School, Hannover, Germany; 5 Department of Laboratory Animal Science, Hannover Medical School, Hannover, Germany

**Keywords:** Neuroinflammation, translocator protein, PET, epileptogenesis, minocycline

## Abstract

Epileptogenesis-associated brain inflammation might be a promising target to prevent or attenuate epileptogenesis. Positron emission tomography (PET) imaging targeting the translocator protein (TSPO) was applied here to quantify effects of different dosing regimens of the anti-inflammatory drug minocycline during the latent phase in two rodent models of epileptogenesis. After induction of epileptogenesis by status epilepticus (SE), rats were treated with minocycline for 7 days (25 or 50 mg/kg) and mice for 5 or 10 days (50 or 100 mg/kg). All animals were subjected to scans at 1 and 2 weeks post-SE. Radiotracer distribution was analyzed and statistical parametric mapping (SPM) was performed, as well as histological analysis of astroglial activation and neuronal cell loss. Atlas-based analysis of [^18^F]GE180 PET in rats revealed a dose-dependent regional decrease of TSPO expression at 2 weeks post-SE. Results of SPM analysis depicted a treatment effect already at 1 week post-SE in rats treated with the higher minocycline dose. In mice, TSPO PET imaging did not reveal any treatment effects whereas histology identified only a treatment-related reduction in dispersion of dentate gyrus neurons. TSPO PET served as an auspicious tool for temporal monitoring and quantification of anti-inflammatory effects during epileptogenesis. Importantly, the findings underline the need to applying more than one animal model to avoid missing treatment effects. For future studies, the setup is ready to be applied in combination with seizure monitoring to investigate the relationship between individual early treatment response and disease outcome.

**Electronic supplementary material:**

The online version of this article (10.1007/s13311-020-00834-5) contains supplementary material, which is available to authorized users.

## Introduction

With 2.4 million people diagnosed with epilepsy worldwide each year, it is one of the most common chronic brain diseases [1]. Despite the availability of numerous anti-epileptic drugs, in approximately 30% of all epilepsy patients, these medications fail to suppress the spontaneously recurring epileptic seizures [2]. Thus, it is desirable to develop therapeutic strategies that target the mechanisms underlying disease development rather than the symptoms of epilepsy [3]. Temporal lobe epilepsy, the most drug-refractory type of epilepsy [4], is frequently preceded by brain insults, which initiate a cascade of changes in the brain resulting in epilepsy development [5, 6]. The period between the initiating insult and the first clinically obvious seizures, termed “latent period,” offers a window of opportunity for anti-epileptogenic treatment. For evaluation of anti-epileptogenic therapies, post-status epilepticus (SE) models are an accepted and widely used tool [7]. However, no epilepsy-preventive therapeutic strategies have been identified to date [8].

Prolonged inflammatory responses of the brain, characterized by sustained microglial and astroglial activation as well as overexpression of pro-inflammatory molecules, have been proposed to play a causative role in epileptogenesis [9, 10]. Evidence of inflammatory processes were revealed by *ex vivo* techniques such as immunohistochemistry or autoradiography not only in several post-SE animal models [11–13] but also in human brain specimens from structural forms of epilepsy with an acquired or genetic etiology as recently reviewed [14]. Moreover, the availability of dedicated small animal positron emission tomography (PET) scanners allows serial quantitative *in vivo* imaging of epileptogenesis-associated inflammatory processes [11], targeting inflammation-related molecules such as the translocator protein (TSPO), which is predominantly expressed by activated microglia [15, 16]. Recently, our group has elucidated the spatiotemporal profile of microglial activation during epileptogenesis both in the lithium-pilocarpine rat model and in the intrahippocampal kainate mouse model by longitudinal TSPO PET imaging and complementary *in vitro* techniques [17, 18]. In these models, microglial activation was evident in epilepsy-associated brain regions such as the hippocampus from 2 days to at least 3 weeks after status epilepticus (SE), reaching its maximum at about 1–2 weeks depending on the brain region. Considering these findings, early anti-inflammatory treatment may represent a promising strategy to counteract epileptogenesis after brain insults [19] and treatment effects may be monitored by *in vivo* imaging of brain inflammation.

Minocycline represents a promising candidate for anti-inflammatory treatment during epileptogenesis [20, 21]. Despite often referred to as an inhibitor of microglial activation, minocycline affects either directly or indirectly also other cells types such as neurons, astrocytes, and oligodendrocytes. The specific mechanism of its anti-inflammatory activity is unknown. Nevertheless, various preclinical studies have revealed both disease-modifying effects on epileptogenesis and/or effects on neuropathological hallmarks of epilepsy like neurodegeneration or neuroinflammation [22–25]. However, *in vivo* assessment of minocycline effects on its supposed main target, i.e., activated microglia, cannot be achieved by using only standard tissue extraction-based techniques. Furthermore, assessing the interaction between early treatment effects and later disease outcome in individual animals is not possible by using histology. To facilitate such investigations, studies validating the utility of preclinical TSPO PET as a non-invasive monitoring tool for anti-inflammatory treatment response during the latent phase are needed.

In the present study, we quantified the anti-inflammatory efficacy of minocycline treatment during the latent period in the pilocarpine rat model and the intrahippocampal kainate mouse model by serial *in vivo* TSPO PET. We complemented PET imaging data by *in vitro* autoradiography using the same radio-ligand as well as histological evaluation of treatment impact on neurodegeneration and astrogliosis.

## Materials and Methods

### Animals

Adult female Sprague-Dawley rats (200–220 g) were purchased from Harlan (Italy). Male NMRI mice were obtained from Charles River Laboratories (Sulzfeld, Germany) at the age of 7 weeks. The choice of sex is based on earlier work of our and the Löscher research group [17, 18, 26, 27]. Despite the use of both sexes is desirable, we decided to stick to the established protocols, resulting in reasonable epileptogenesis induction rates with low mortality, as it has been shown before that the change of sex, strain, or breeder can significantly influence epileptogenesis characteristics [26–28]. Rats were housed in pairs whereas mice were housed in groups under controlled climate and controlled hygienic conditions in individually ventilated cages under a 14/10-h light-dark cycle. They received autoclaved tap water and standard laboratory chow (Altromin 1324, Lage, Germany) ad libitum. Animals were allowed to adapt to the new conditions for at least 1 week and were handled repeatedly before the experiments started. Experiments were formally approved by the responsible local authority and were conducted in accordance with European Communities Council Directives 86/609/EEC and 2010/63/EU. All efforts were made to minimize pain, suffering, and the number of animals. The principles outlined in the ARRIVE guidelines and the Basel declaration including the 3R concept have been considered.

### General Study Design

Animals were randomly assigned to experimental groups. Subsequent to SE induction by pilocarpine or intrahippocampal kainate injection, animals underwent either minocycline or vehicle treatment. [^18^F]GE180 PET/CT scans were performed 1 and 2 weeks after SE induction. Scan time points were selected based on findings previously reported by our group [17, 18]. In a recent study, the latency phase in the pilocarpine rat model was found to range from 6 to 10 days post-SE (mean 7 days) [29]. The latency phase for male NMRI mice in the intrahippocampal mouse model was found to be 10–14 days post-SE [26]. Therefore, in average, the first imaging time point lays within the latency phase whereas the second represents the early disease phase. After the second scan, all animals were euthanized for further analysis of brain slices by *in vitro* autoradiography and immunohistochemistry. Unless stated otherwise, all chemicals used were of analytic grade and purchased from Sigma-Aldrich (Steinheim, Germany).

### Post-SE Models of Epileptogenesis

SE in rats and mice was induced as described elsewhere [17, 18]. Shortly, 14–16 h after pretreatment with lithium chloride (127 mg/kg p.o.) and 30 min after pretreatment with methyl-scopolamine (1 mg/kg i.p.), rats (*n* = 34) received rationed i.p. injections of pilocarpine (max. 60 mg/kg). Behavioral seizures were assessed according to Racine’s scale [30]. Convulsive SE was interrupted after 90 min by diazepam (Ratiopharm GmbH, Ulm, Germany, max. 25 mg/kg i.p.). Mice (*n* = 36) were anesthetized with choral hydrate (500 mg/kg, i.p.). Kainate monohydrate (0.21 μg in 50 nL saline) was injected over 60 s into the right cornu ammonis subregion 1 (CA1) of the dorsal hippocampus. The average time under chloral hydrate anesthesia was 124 ± 42 min. Kainate injection took place about 40 min after begin of anesthesia. Mice were closely observed for at least 2 h for clinical signs of SE upon awakening from anesthesia and 1 mouse died shortly after SE. Typically, every mouse shows SE symptoms within this period, which was also the case in this study. SE was not interrupted in mice. In the days following SE, animals were closely monitored, received mashed laboratory chow, and were injected with glucose electrolyte solution (Sterofundin HEG-5, Braun) until resuming normal feeding behavior. Animals resumed normal feeding within 2.76 ± 2.90 and 3.57 ± 0.76 days post-SE for mice and rats, respectively.

### Minocycline Treatment

Minocycline (minocycline hydrochloride; Tokyo Chemical Industry) was freshly dissolved in saline and administered i.p. in a volume of 2 ml/kg in rats and 10 ml/kg in mice. Rats received either 25 (*n* = 7) or 50 mg/kg (*n* = 5) daily for 7 days starting 24 h post-SE. Mice received either 50 mg/kg once daily over 5 days (*n* = 7) or 50 mg/kg twice daily over 10 days (*n* = 9), both starting 6 h after SE. Vehicle treatment was performed with saline instead of minocycline solution (mice *n* = 19; rats *n* = 9). Dosing regimens were derived from earlier studies in mice and rats. In the pilocarpine-induced SE, rat model reduced microglial activation, reduced production of IL-1ß and TNFα, and prevention of neuronal cell loss was observed after treatment with 45 mg/kg minocycline once daily for 14 days. Further, attenuation of spontaneous recurrent seizure development indicating disease-modifying properties regarding epilepsy development was described [25]. In mice, a pronounced reduction in microglia activation after systemic kainate-induced SE at postnatal day 25 was mediated by minocycline treatment over 6 days (20 mg/kg) [22]. The dosing regimens were adapted to stop the treatment before the first PET scan. As the 50 mg/kg dose resulted in reduced microglia activation in rats, we started with this dose in the mouse model.

### PET Imaging

PET imaging and the synthesis of [^18^F]GE180 was performed as described before [17, 18] with a resulting specific activity from 444.8 to 588.6 GBq/μmol. Due to logistical limitations it was not possible to scan all animals at all time points. Under isoflurane anesthesia, animals were placed prone in an imaging chamber with the brain at the center field of view of a dedicated small animal PET/CT camera (Inveon PET scanner, Siemens). [^18^F]GE180 (23.32 ± 4.25 MBq in rats, 12.85 ± 0.66 MBq in mice) was injected into a lateral tail vein. In rats, dynamic data were acquired over 1 h in 32 frames as described before [18]. In mice, a static 20-min PET acquisition was performed after a 40-min uptake period under anesthesia, each followed by a low-dose CT scan. For reconstruction of images, an iterative ordered subset expectation maximization three-dimensional/maximum posteriori (OSEM3D/fastMAP) algorithm with standard corrections including transmission correction was applied.

Image analysis and kinetic modeling were completed using PMOD software (PMOD 3.7, Zürich, Switzerland). For co-registering PET images to an MRI template [31, 32], CT images were matched to both the corresponding PET images and the MRI template. Subsequently, a volume of interest (VOI) template based on the MRI atlas was applied to the co-registered images [33, 34]. [^18^F]GE180 uptake was quantified as percent injected dose per gram tissue (%ID/g) in hippocampus, thalamus, and piriform cortex in rats or whole cortex in mice.

For kinetic modeling of PET data gained in rats, VOIs (16.0 mm^3^, cuboid) were defined for the left and right carotid artery in the dynamic images and averaged in order to generate time activity curves of whole blood [35, 36]. Using these image-derived input functions, time activity curves were fitted to a two-tissue compartment model [37] in order to obtain the volume of distribution (V_t_). Region-based Logan graphical analysis was additionally performed (data not shown) and both methods provided similar results. Additionally, V_t_ maps were calculated in PMOD by voxel-wise modeling using the Logan plot.

Calculation of averaged images and statistical parametric mapping (SPM) was performed using MATLAB software (The MathWorks) and SPM12 (University College London). For SPM analysis, differences between vehicle and minocycline-treated animals were calculated by unpaired 2-sample *t* tests setting a significance level threshold of 0.01 (uncorrected for multiple comparisons). The minimum cluster size of voxels was set to 100 for rats and to 50 for mice.

### Autoradiography and Immunohistology

After the last PET/CT scan, animals were euthanized, and brains were rapidly removed, covered with Tissue-Tek® (Sakura® Finetek, USA), snap-frozen, and stored at − 20 °C. Coronal 14-μm-thick slices were cut in a cryostat (Microm HM560; Schwerte, Germany) and mounted on Histobond slides (Marienfeld, Germany). Rat brains were sliced at section levels − 3.6 and − 5.2 mm relative to bregma [38] and mouse brains at − 1.82 and − 2.92 mm relative to bregma [39], in order to include thalamus, hippocampus and (piriform/entorhinal) cortex as regions for tissue analysis. The administration of radiotracers for PET imaging before killing the animals does not interfere with later performed autoradiography or immunohistochemistry, as the used radiotracers are administered only in nano- to picomolar concentrations, and are therefore much below target saturation.

Autoradiography was performed as described earlier [18]. Brain sections were incubated with [^18^F]GE180 (~ 2 MBq per 100 ml PBS), exposed to a high-resolution phosphor imaging plate (PerkinElmer), and digitized using Cyclone scanner (PerkinElmer). Images were co-registered to the 3D VOI atlas, using PMOD software. To calculate activity concentration (Bq/mm^2^), gray scale values were interpolated to a calibration curve (10–500 kBq/ml).

As increased TSPO PET signals were confirmed by immunohistochemistry of activated microglia during epileptogenesis before [18, 40], here, we focused on immunohistochemical staining of astrocytes, for which TSPO expression under certain conditions has also been shown [40]. Furthermore, neurodegeneration was investigated as a marker for potential neuroprotective minocycline effects. Brain slices, fixated with 4% paraformaldehyde, were immunostained for the astrocyte marker glial fibrillary acidic protein (polyclonal rabbit anti-GFAP antibody, Dako), or the neuronal marker neuronal nuclear antigen (NeuN; mice, #ABN78 Millipore; rats, MAB377) applying a previously described procedures [18, 41]. Slices were incubated with Vectastain ABC reagent (Biozol) and subsequently stained by nickel-intensified diaminobenzidine reaction.

The severity of neuronal damage and astrocyte activation was assessed in hippocampus and thalamus in both slicing levels mentioned above and values averaged afterwards. Sub-regions of the hippocampus were scored separately and values averaged afterwards. For scoring of neurodegeneration in NeuN-stained sections, a semi-quantitative system was adapted from Polascheck et al. [42]: 0, no obvious neuronal cell loss; 1, < 20% neuronal cell loss; 2, 20–50% neuronal cell loss; 3, > 50–75% neuronal cell loss; 4, > 75% neuronal cell loss. In the same slices, dispersion of the dentate gyrus (DG) neurons was scored: 0, no dispersion; 1, slight dispersion, cells are packed less densely; 2, moderate dispersion, gaps between cells; 3, severe dispersion, big gaps between cells; 4, very severe dispersion, many cells are separated from each other. Additionally, the number of neurons in the dentate hilus was quantified in rat brain slices using AxioVision software (Zeiss, Jena, Germany) as described before [42]. Astroglia activation score values were defined as: 0, resting, < 10% activated cells; 1, mostly resting, approximately 30% activated cells; 2, approximately 60% activated cells, some resting; 3, > 90% activated cells, densely packed [18]. Every analysis was performed at 100-times-magnification (Leica, Wezlar, Germany) and investigators were blinded to experimental groups. Slices were chosen randomly. For autoradiography, two (rats) or three (mice) slices per animal and section level were analyzed and the mean was calculated. For histology, one slice per animal, staining and section level was assessed. Mean of left and right hemisphere was calculated (only rats) followed by calculation of the mean of section levels (mice and rats).

### Statistical Analysis

Statistical analyses was performed using Prism7 (GraphPad Software, La Jolla, CA, USA) software. All data are presented as mean ± standard deviation (SD). Values of *p* < 0.05 were considered statistically significant. For data of rats post-SE, one-way analysis of variance (ANOVA) with Dunnett’s post hoc test for multiple comparisons was used to test for differences between minocycline- and vehicle-treated rats at the same time point in [^18^F]GE180 V_t_, uptake, and *in vitro* binding, as well as hilar neuronal density. Non-parametric scoring data of histological analyses were compared using the Kruskal-Wallis test, followed by Dunn’s post hoc test for multiple comparisons of minocycline- *versus* vehicle-treated groups. For data of mice, Student’s *t* test was applied to compare [^18^F]GE180 uptake in minocycline- and vehicle-treated groups. Results of histological analyses were assessed using Mann-Whitney test. Correlations of *in vivo* PET data and *in vitro* autoradiography were calculated by Pearson’s linear regression analysis. Required minimal group sizes for both animal models (*n* = 6) were estimated using power analysis (G*Power, Kiel University, Germany; two-tailed unpaired *t* test, power: 0.8, α-error: 0.05, effect size: 2.0). If possible, additional animals were included to account for a certain drop-out rate.

## Results

### Lithium-Pilocarpine Post-SE Rat Model

Thirty out of 34 rats reached SE (overall induction rate 88%), requiring an average pilocarpine dose of 35.3 ± 5.1 mg/kg. Rats which did not develop SE were excluded from all further experiments. Within the first 72 h after SE 8 animals died (mortality rate 26.7%). In addition, one animal of the low-dose minocycline group died 6 days after SE.

In a previous study in the pilocarpine rat model, we already determined the SE-induced magnitude of TSPO increase in comparison to baseline data by [^11^C]PK11195 PET [18]. After SE, [^11^C]PK11195 uptake was elevated in most brain regions, including those associated with epileptogenesis (hippocampus, piriform cortex, thalamus) but not the cerebellum. The increase in TSPO binding reached its peak approximately 7 days after SE (up to 2.08-fold increase in the ventral hippocampus) and remained significantly above baseline values up to 3 weeks after SE.

**Fig. 1:**
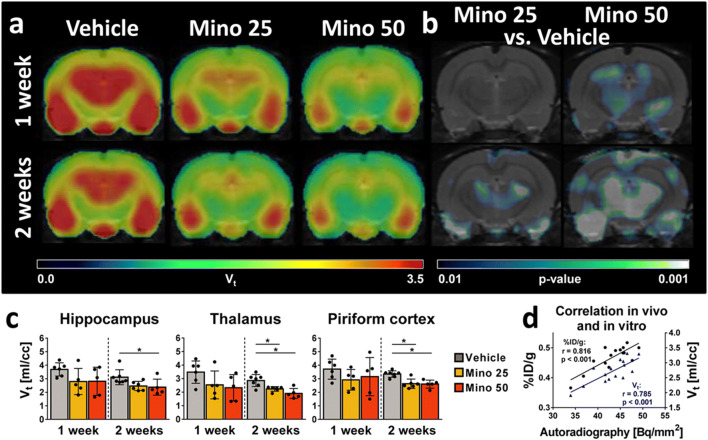
Analysis of [^18^F]GE180 *in vivo* brain PET and *in vitro* autoradiography data in vehicle- and minocycline-treated rats after pilocarpine-induced status epilepticus (SE). (**a**) Averaged coronal [^18^F]GE180 volume of distribution (V_t_) maps [ml/cc] at the level of maximal TSPO PET signal (~ 3.6 mm caudal to bregma) 1 and 2 weeks after SE in vehicle- and minocycline-treated rats (25 mg/kg or 50 mg/kg for 7 days). (**b**) Corresponding statistical parametric mapping (SPM) analysis comparing minocycline-treated *versus* vehicle-treated rats post-SE (*t* test, *p* < 0.01, minimum cluster size of 100 voxels). (**c**) Atlas-based analysis of [^18^F]GE180 V_t_ in epileptogenesis-associated brain regions. Data are mean ± SD. Significant group differences calculated by one-way ANOVA and Dunnett’s post hoc test are indicated by asterisks (*p* < 0.05). (**d**) Pearson correlation analysis of [^18^F]GE180 *in vitro* binding [Bq/mm^2^] to *in vivo* uptake [%ID/g] (indicated in black) and V_t_ [ml/cc] (indicated in blue). Mino, minocycline

Effects of minocycline treatment on epileptogenesis-associated microglial activation were evaluated by repeated [^18^F]GE180 PET imaging of TSPO expression. Atlas-based [^18^F]GE180 uptake analysis did not reveal significant differences between groups, except for a decrease of TSPO expression 2 weeks after SE in the thalamus of rats treated with 50 mg/kg minocycline in comparison to vehicle-treated rats (*p* = 0.023, Table 1). However, we observed significant reduction of TSPO expression in several brain regions when comparing [^18^F]GE180 volume of distribution (V_t_) between vehicle- and minocycline-treated rats (Fig. 1a, c). At 1 week post-SE, no changes could be identified in the low-dose (25 mg/kg) minocycline-treated animals, neither in the atlas-based nor in the SPM analysis. High dose (50 mg/kg) minocycline-treated rats showed no decrease considering only the atlas-based analysis, but SPM identified decreased voxels in the hippocampus and the thalamus (Fig. 1b). At 2 weeks post-SE, V_t_ was significantly lower for both minocycline doses. In rats treated with 25 mg/kg minocycline, V_t_ was up to 22% lower in the thalamus than in vehicle-treated rats (*p* = 0.012). V_t_ in rats treated with 50 mg/kg minocycline was even more decreased, reaching maximal reduction also in the thalamus (−36%, *p* < 0.001). SPM confirmed the atlas-based analysis 2 weeks post-SE (Fig. 1b).

**Table 1:** *In vivo* brain uptake and *in vitro* autoradiography of [^18^F]GE180 in rats after lithium-pilocarpine-induced SE

		Hippocampus		Thalamus		Piriform cortex	
		Mean ± SD	*p* value	Mean ± SD	*p* value	Mean ± SD	*p* value
Uptake 1 week post-SE [%ID/g]	Vehicle	0.60 ± 0.08		0.57 ± 0.11		0.60 ± 0.11	
	Mino 25	0.53 ± 0.13	0.434	0.47 ± 0.11	0.252	0.56 ± 0.13	0.792
	Mino 50	0.52 ± 0.08	0.328	0.44 ± 0.10	0.132	0.57 ± 0.11	0.814
Uptake 2 weeks post-SE [%ID/g]	Vehicle	0.47 ± 0.05		0.46 ± 0.05		0.50 ± 0.03	
	Mino 25	0.43 ± 0.08	0.398	0.40 ± 0.07	0.192	0.46 ± 0.05	0.328
	Mino 50	0.45 ± 0.04	0.681	0.37 ± 0.03	0.023	0.50 ± 0.08	0.999
*In vitro* autoradiography 2 weeks post-SE [Bq/mm^2^]	Vehicle	42.89 ± 3.26		39.06 ± 1.34		49.14 ± 4.86	
	Mino 25	42.46 ± 1.66	0.955	36.80 ± 2.00	0.733	47.66 ± 3.71	0.079
	Mino 50	41.66 ± 1.57	0.701	33.94 ± 1.31	0.018	41.63 ± 3.00	0.001

*SE*, status epilepticus; *Mino*, minocycline

*In vitro* autoradiography of brain slices generated from rats at 14 days after SE induction (Table 1) also revealed decreased [^18^F]GE180 *in vitro* binding in minocycline-treated animals compared to the vehicle-treated group. This reached significance in the thalamus (13% reduction, *p* = 0.018) and the piriform cortex (15% reduction; *p* = 0.001) in rats receiving 50 mg/kg minocycline, although there was a trend for reduced [^18^F]GE180 binding in the piriform cortex in rats treated with the lower minocycline dose (*p* = 0.079). To confirm validity of the *in vivo* data, correlation analysis between *in vitro* and *in vivo* data was performed, and [^18^F]GE180 binding showed a very strong correlation with both *in vivo* uptake (*r* = 0.816, *p* < 0.001) and V_t_ (*r* = 0.785, *p* < 0.001).

**Fig. 2:**
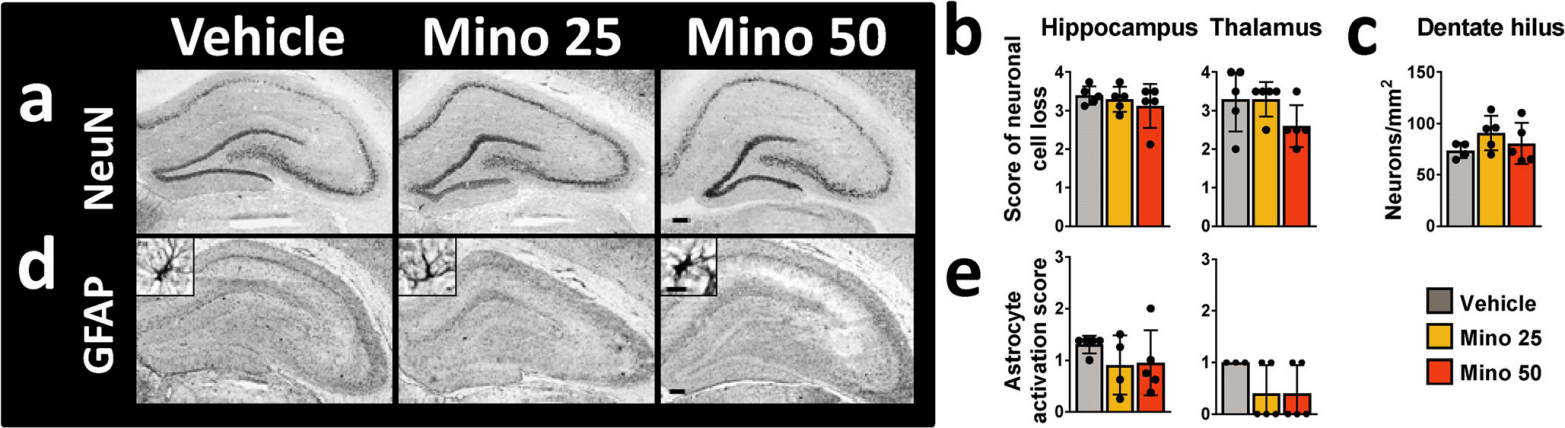
Histological analysis of neurodegeneration and astroglial activation in brain slices of vehicle- and minocycline-treated rats 2 weeks after pilocarpine-induced status epilepticus. Representative images of (**a**) neuronal nuclear antigen (NeuN)- or (**d**) glia acidic fibrillary protein (GFAP)-immunostained brain slices (scale bars: 500 μm for overview and 10 μm for detailed images). Scoring of (**b**) neuronal cell loss and (**e**) astrocyte activation in selected brain regions. (**c**) Density of hippocampal hilus neurons per mm^2^. All data are mean ± SD. *Mino*, minocycline

Brain slices of rats 2 weeks after SE were immunostained for neurons (NeuN) and astrocytes (GFAP; Fig. 2a, d). Previously published comparisons with naïve rats revealed clear glial activation as well as neurodegeneration in rats 2 weeks post-SE [18]. However, scoring of neuronal cell loss and astrocyte activation did not reveal any treatment effects (Fig. 2b, e). This was also true for the neuronal density in the hippocampal dentate hilus (Fig. 2c).

### Intrahippocampal Kainate Post-SE Mouse Model

All animals undergoing intrahippocampal kainate injections showed typical signs of SE (circling, head tilt, head nodding, chewing, mild convulsions of forelimbs). One minocycline-treated mouse (50 mg/kg once daily) died during PET scanning at 1 week after SE. Mice treated twice daily with 50 mg/kg minocycline developed severe diarrhea within several days. Therefore, this treatment protocol did not meet the criterion of acceptable tolerability and mice were excluded from all further experiments.

**Fig. 3:**
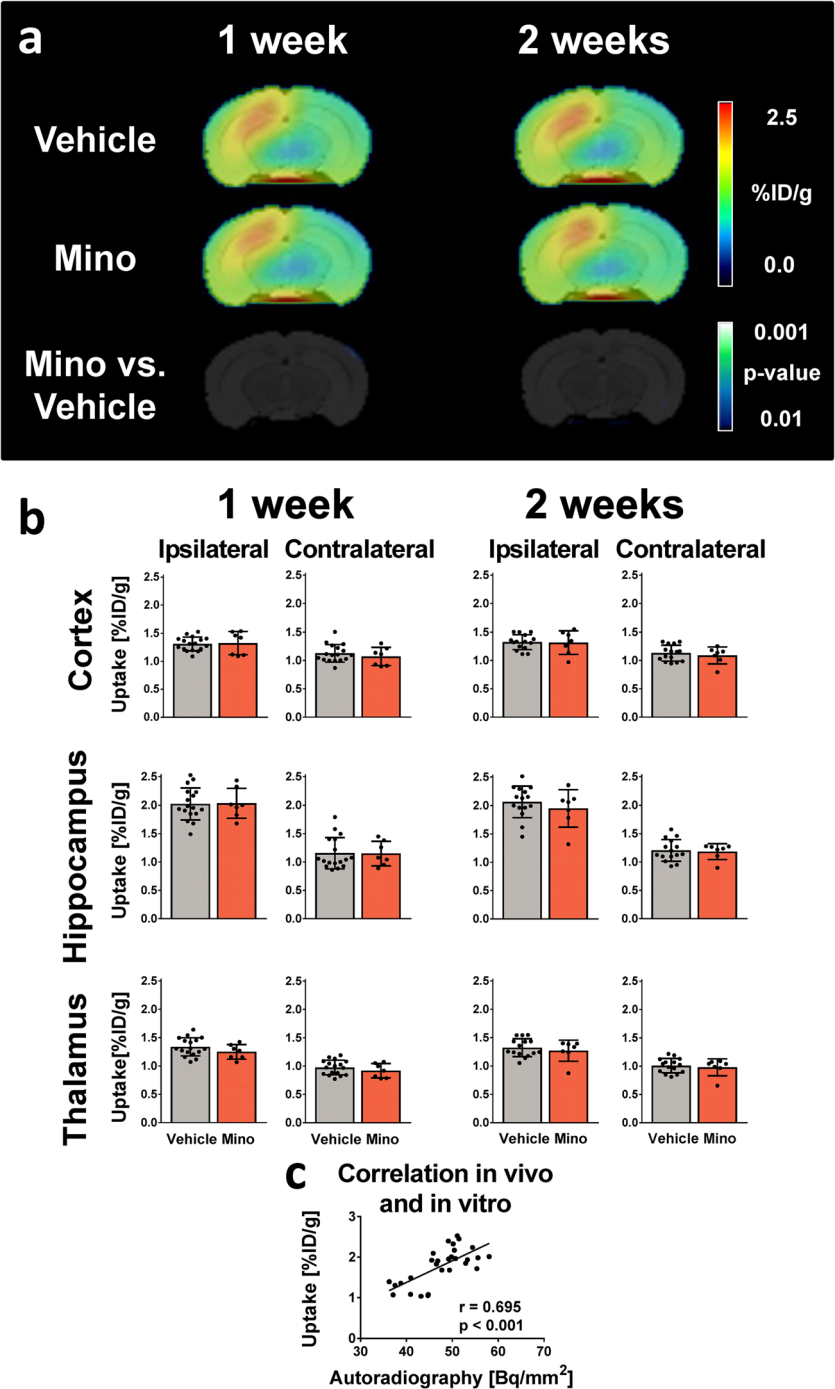
Analysis of TSPO brain PET in vehicle- and minocycline-treated mice after status epilepticus (SE) induced by intrahippocampal kainate injection in mice. (**a**) Averaged [^18^F]GE180 uptake images [%ID/g] of vehicle- and minocycline-treated mice (50 mg/kg for 5 days) 1 and 2 weeks after SE at the level of maximal TSPO PET signal (2.92 mm caudal to bregma). Bottom row: Statistical parametric mapping (SPM) analysis comparing minocycline- and vehicle-treated animals (*t* test, *p* < 0.01, minimum cluster size of 50 voxels). (**b**) Atlas-based analysis of [^18^F]GE180 uptake in selected brain areas. Data are mean ± SD. (**c**) Pearson correlation analysis of [^18^F]GE180 *in vitro* binding [Bq/mm^2^] to *in vivo* uptake [%ID/g]. *Mino*, minocycline

Mice underwent [^18^F]GE180 PET scans at 7 and 14 days after SE induction (Fig. 3). In a previous study, at around 1 week after SE, a distinct elevation of TSPO signal became apparent in the ipsilateral dorsal hippocampus (2.17-fold), but also in the ipsilateral ventral hippocampus (1.64-fold), thalamus (1.58-fold), and cortex (1.44-fold). At 14 days post-SE, this increase was less distinct but still statistically significant [17]. Minocycline treatment did not alter [^18^F]GE180 uptake compared to vehicle treatment in the analyzed VOIs at any of the time points studied (Fig. 3a, b). This was in line with the results of the SPM analysis which did not reveal significantly different voxels (Fig. 3a) and autoradiography (data not shown). *In vivo* data showed a strong correlation with *in vitro* autoradiography (*r* = 0.695, *p* < 0.001, Fig. 3c).

**Fig. 4:**
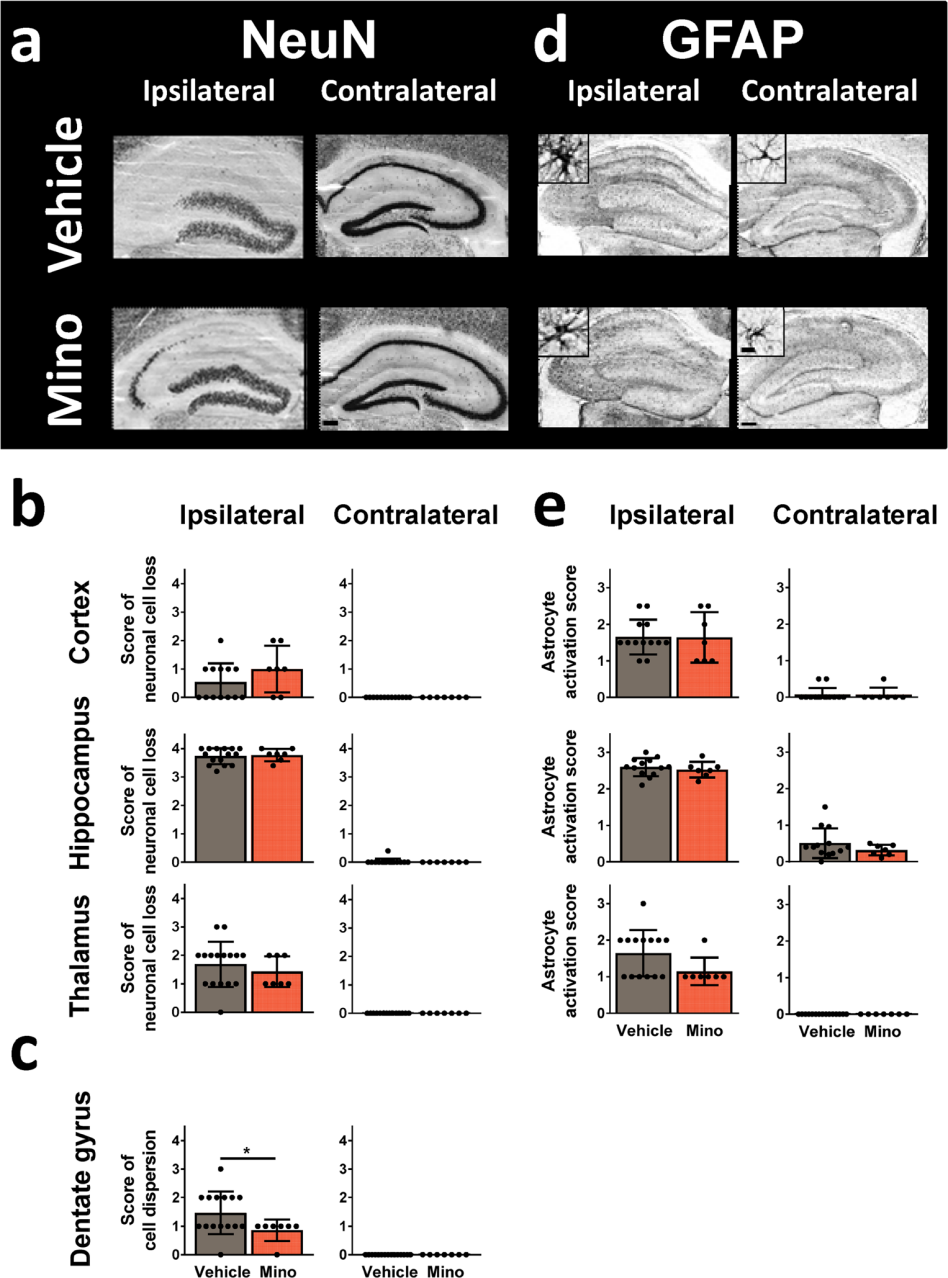
Histologic analysis of neurodegeneration and astroglial activation in in brain slices of vehicle- and minocycline-treated mice 2 weeks after status epilepticus induction by intrahippocampal kainate injection. Representative images of (**a**) neuronal nuclear antigen (NeuN)- or (**d**) glia acidic fibrillary protein (GFAP)-immunostained brain slices (scale bars: 500 μm for overview and 10 μm for detailed images). Scoring of (**b**) neuronal cell loss, (**c**) neuronal cell dispersion, or (**e**) astrocyte activation in selected brain regions. Data are mean ± SD. Significant group difference calculated by Mann-Whitney test is indicated by asterisk (*p* < 0.05)

Astrocyte activation, neurodegeneration, and neuronal cell dispersion was assessed in brain slices from mice 15 days after surgery (Fig. 4). Ipsilateral, vehicle-treated mice showed high score values for all parameters in all investigated brain areas. No impact of minocycline on neurodegeneration or astrocyte activation was observed (Fig. 4b, e). Nevertheless, neuronal cell dispersion in the dentate gyrus was reduced in minocycline-treated mice (*p* = 0.040; Fig. 4c).

## Discussion

In this study, we demonstrate by molecular *in vivo* imaging that minocycline treatment significantly reduces TSPO expression after pilocarpine-induced SE in rats, although it has no TSPO-reducing effect in the intrahippocampal kainate mouse model.

TSPO is primarily expressed on activated microglia and therefore allows *in vivo* evaluation of ongoing inflammatory processes [16]. Although TSPO might be expressed also in other brain cell types like astrocytes under certain conditions, there is no evidence that this is significantly the case during early epileptogenesis [33]. In PET studies, we have shown before that TSPO expression is substantially enhanced in epileptogenesis-associated brain regions within the first 2 weeks after SE in both applied animal models, which guided imaging time points and treatment duration for the present study [17, 18].

Minocycline, originally known for its anti-microbial mode of action, has been shown to attenuate the immune response via various pathways [20]. It reduces the production of pro-inflammatory cytokines and matrix metalloproteinases [21] and in an amyotrophic lateral sclerosis mouse model, it inhibited microglia to develop a more pro-inflammatory phenotype [43]. Furthermore, an inhibiting effect of minocycline on astrocytes, oligodendrocytes, neurons, and T cells has been reported [44, 45]. Also a number of studies in rat and mouse models of epileptogenesis revealed effects both on neuroinflammation and the development of seizure susceptibility. In a two-hit SE mouse model, minocycline treatment after the first SE early in life revoked the increased susceptibility to the second SE later in life and reduced microglia activation [22]. Mice subjected to intrahippocampal kainate injection and minocycline treatment showed decreased neurodegeneration [23]. Minocycline has also been shown to delay kindling acquisition in amygdala-kindled rats using a dose of 25 mg/kg [24]. Moreover, prolonged minocycline treatment following pilocarpine-induced SE in rats inhibited microglial activation assessed by immunohistochemistry during epileptogenesis, and reduced the frequency, duration, and severity of spontaneous recurrent seizures in the chronic phase of epilepsy [25].

Thus, the observed reduction of [^18^F]GE180 signal in the pilocarpine rat model generally lines up with prior published effects of minocycline. Nevertheless, in the study of Wang et al. [25], a dose of 45 mg/kg minocycline administered daily for 14 days, beginning immediately after SE, inhibited microglial activation almost completely and exerted a disease-modifying effect. In the present study, the reduction of microglia activation was less intense and no attenuation of neurodegeneration or astrocyte activation was observed which may be due to the later start and shorter duration of the treatment. Although group size was based on power analysis, it seems that the actual data variation was bigger than estimated. Therefore, increasing the sample size might have resulted in significances, e.g., for TSPO signal reduction 1 week after SE (Fig. 1c). Minocycline has certain anti-convulsive effects which have been shown in animals as well as in a human case study [46, 47]. In line with this idea, a single minocycline injection in rats (25 mg/kg) given shortly after interruption of pilocarpine-induced SE found a certain degree of hippocampal neuroprotection [48]. Thus, SE might have been attenuated in the study by Wang et al. [25] as a consequence of direct minocycline administration causing less severe inflammation from the beginning. We chose a treatment start of 24 h after SE both to eliminate anti-convulsive and insult-modifying effects of minocycline. In addition, complete suppression of SE-induced neuroinflammatory response might also have harmful consequences [49]. In this regard, direct anti-inflammatory corticosteroid treatment after interruption of pilocarpine-induced SE in rats has shown deteriorating rather than beneficial effects before [50]. Interestingly, a recent study by Russmann et al. [51] found only very limited minocycline effects on microglia activation or neurodegeneration using a different dosing scheme over 2 weeks after electrically induced SE in rats. Nevertheless, in this study, minocycline treatment had a positive impact on spatial learning, hyperactivity, and hyperlocomotion, whereas seizure development was not attenuated. Further, a study by Kwon et al. found no neuroprotective action when treating young rats with minocycline immediately after a double-hit insult (pilocarpine-mediated SE and lipopolysacharide injection) [52].

As the 50 mg/kg dose was more efficient than 25 mg/kg in rats after pilocarpine-induced SE, it was transferred to the mouse model. However, in the intrahippocampal kainate mouse model, TSPO PET did not detect any treatment-related changes. This is very unlikely be caused by a too low dose. First, applying a higher dose caused severe side effects, causing the termination of this experimental group. Second, a dose of 20 mg/kg minocycline injected once daily over 7 days in a mouse model of SE induced by systemic kainate administration during early life, decreased microglial activation to a pronounced extent [22]. This discrepancy to our findings may be attributed to differences in animal age or kainate administration route. It is also possible that in this context, the sensitivity of TSPO PET is not sufficient to reveal reduced neuroinflammation. However, minocycline treatment after focal cerebral ischemia in rats reduced uptake of the TSPO tracer [^18^F]DPA-714 in the infarcted area [53]. In addition, in two other studies in the intrahippocampal kainate mouse model, we detected a reduced TSPO PET signal after anti-inflammatory treatment with curcumin or fingolimod 2 weeks after SE (unpublished data), confirming the general suitability of the chosen approach. In line with the observation that minocycline impacted epileptogenesis-related changes in rats, although this was not the case in mice, a study in neonatal rats and mice subjected to hypoxic-ischemic encephalopathy reported similar findings. Minocycline attenuated brain injury only in rats but even augmented it in mice, which may point towards a species-dependent addressability to minocycline [54]. Morphological evaluation of astrocyte activation did not reveal significant treatment effects. It has to be considered, however, that sole morphological analysis might not account for all anti-inflammatory effects on astrocytes.

Analysis of V_t_ revealed more pronounced treatment effects than analysis of [^18^F]GE180 uptake in rats. These results need to be considered because for mice only uptake analysis was performed. Due to the repetitive PET scanning ruling out blood-sampling, the lack of a sufficient cerebral reference region and the small size of blood vessels combined with spill in from the surrounding hampering the generation of image-derived input functions, we could not establish a way to analyze [^18^F]GE180 kinetics in mice. The observation that V_t_ can be more sensitive than uptake analysis was made before and can be explained by the fact that tracer uptake is influenced by various potential confounders such as tissue perfusion or metabolism, whereas kinetic analysis accounts for these [36]. This is also generally in line with a TSPO PET imaging study in TLE patients showing more pronounced V_t_ differences, but also higher variation, compared to standardized uptake values [55]. Nevertheless, in line with earlier investigations [17, 18], the strong correlation of *in vivo* tracer uptake with the *in vitro* autoradiography data shown here for data from both rats and mice supports the application of uptake analyses.

Although the focus of this TSPO PET study was assessing early anti-inflammatory effects of minocycline treatment, other studies performed a follow up of rats after SE to the chronic disease phase: Bertoglio et al. conclude from their findings that TSPO PET might serve as a biomarker for early prediction of epileptic seizure frequency [56], and a further study identified TSPO PET as a predictor of treatment response to anti-seizure drugs [57]. These findings support the idea that TSPO PET might also be suitable for early prediction of response to anti-epileptogenic or disease-modifying therapies; however, this remains to be assessed in future studies. In the present study TSPO PET identified minocycline-mediated reduction of neuroinflammation during epileptogenesis in the lithium-pilocarpine rat model, but not in the intrahippocampal kainate mouse model of epileptogenesis. It has to be considered that minocycline’s anti-epileptogenic properties might be mediated by further mechanisms beyond a reduction of microglia activation. Nonetheless, the described setup can now be used to preclinically investigate individual treatment responses during the latent phase and their correlation to chronic disease outcome. Furthermore, PET may be utilized to identify purposeful drug doses and treatment durations. TSPO PET might also be a valuable tool for evaluation of non-anti-inflammatory treatments preventing secondary neuroinflammation (and therefore increased TSPO expression). In addition, tracers and imaging protocols are very similar for patients, facilitating translation of findings to the clinic for future personalized treatment approaches.

## Electronic Supplementary Material

ESM 1 (PDF 43 kb)
